# A standardized autopsy procurement allows for the comprehensive study of DIPG biology

**DOI:** 10.18632/oncotarget.3374

**Published:** 2015-01-24

**Authors:** Madhuri Kambhampati, Jennifer P. Perez, Sridevi Yadavilli, Amanda M. Saratsis, Ashley D. Hill, Cheng-Ying Ho, Eshini Panditharatna, Melissa Markel, Roger J. Packer, Javad Nazarian

**Affiliations:** ^1^ Research Center for Genetic Medicine, Children's National Health System, Washington, DC, USA; ^2^ Division of Pediatric Neurosurgery, Ann & Robert H. Lurie Children's Hospital of Chicago, Chicago, IL, USA; ^3^ Department of Neurological Surgery, Northwestern University Feinberg School of Medicine, Chicago, IL, USA; ^4^ Division of Pathology, Children's National Health System, Washington, DC, USA; ^5^ Department of Neuro Oncology, Riley hospital for Children, Indiana University Health, Indianapolis, IN USA; ^6^ Brain Tumor Institute, Center for Neuroscience and Behavioral Medicine, Children's National Health System, Washington, DC, USA; ^7^ Institute for Biomedical Sciences, George Washington University, Washington, DC, USA; ^8^ Department of Integrative Systems Biology, George Washington University School of Medicine and Health Sciences, Washington, DC, USA

**Keywords:** Diffuse Intrinsic Pontine Glioma (DIPG), Brainstem Glioma, Autopsy, Histone 3, Orthotopic Injection

## Abstract

Diffuse intrinsic pontine glioma (DIPG) is one of the least understood and most deadly childhood cancers. Historically, there has been a paucity of DIPG specimens for molecular analysis. However, due to the generous participation of DIPG families in programs for postmortem specimen donation, there has been a recent surge in molecular analysis of newly available tumor specimens. Collaborative efforts to share data and tumor specimens have resulted in rapid discoveries in other pediatric brain tumors, such as medulloblastoma, and therefore have the potential to shed light on the biology of DIPG. Given the generous gift of postmortem tissue donation from DIPG patients, there is a need for standardized postmortem specimen accrual to facilitate rapid and effective multi-institutional molecular studies.

We developed and implemented an autopsy protocol for rapid procurement, documenting and storing these specimens. Sixteen autopsies were performed throughout the United States and Canada and processed using a standard protocol and inventory method, including specimen imaging, fixation, snap freezing, orthotopic injection, or preservation. This allowed for comparative clinical and biological studies of rare postmortem DIPG tissue specimens, generation of *in vivo* and in vitro models of DIPG, and detailed records to facilitate collaborative analysis.

## INTRODUCTION

Diffuse intrinsic pontine glioma (DIPG) is one of the most poorly understood childhood cancers. Due to the neuroanatomical location of DIPG and its infiltrative nature, this tumor is not amenable to surgical resection. The lack of readily available surgical specimens, coupled with the progressive nature and high morbidity of this disease has led to a paucity of specimens for molecular studies. However, after decades of stagnation in the study of DIPG biology there has been a recent surge in molecular profiling of DIPG tumor tissue, resulting in identification of novel mutations and genomic aberrations in this lethal cancer [1-5]. One of the main factors contributing to this expanding knowledge of DIPG biology is the selfless gift from children with DIPG and their families of postmortem tumor donation for biological studies. However, despite some efforts to implement a standardized autopsy protocol for specimen acquisition [6], there is a continued need to improve postmortem procurement procedures to ensure optimized use of these precious specimens across North America and Europe, particularly within DIPG collaborative groups and consortia.

Here, we describe an autopsy protocol developed at our institution that encompasses autopsy procedure coordination and the acquisition, processing and storage of frozen, fixed, and fresh tissue specimens for molecular analyses, cell culture and orthotopic injection. We also describe an alternative approach for cases in which assistance with specimen procurement and processing can be conducted in the absence of a trained pathologist. We demonstrate that a standardized approach to specimen acquisition and processing can facilitate identification and characterization of tumor tissue, resulting in successful generation of *in vivo* models of DIPG for further study.

## RESULTS

### Procured DIPG Specimens and Related Clinical Characteristics

A total of 19 brain tumor patients or their families expressed interest in postmortem tissue donation from 2010-2014. Of these, 16 (84%) underwent the procurement procedure. Patient age ranged from 5 to 12 years and autopsy was performed from 5 to 48 hours from the time of death. In 12 (73%) cases, the request for autopsy was initiated by the patient's family, while the remainder of subjects were approached by their neurooncologist for possibility of specimen donation. Request for consent to autopsy was made after radiation treatment when the subjects had stable symptoms and disease burden. A total of nine autopsies were conducted locally, while five were performed at an outside donor institution, and two performed at our institution following whole brain shipment from the donor site.

### Inclusion Criteria

Inclusion criteria were as follows: i) patients of any age with clinical and radiologic diagnosis of diffuse intrinsic pontine glioma, ii) patients with high-grade gliomas originating in the brainstem, iii) patients with focal gliomas of the brainstem.

### Brain Autopsy Procedure

Processing and handling of postmortem DIPG specimens greatly affects the feasibility and results of downstream molecular analyses. Rapid processing of specimens is essential to ensure preservation of high quality mRNA, DNA, protein, and other biologically relevant molecules. One of the major factors contributing to rapid specimen procurement is the availability of a local pathologist, and a suitable autopsy facility for specimen procurement.

However, we have often encountered situations where either a pathologist was not available or coordinating with a pathology team was not possible due to lack of time. Thus, two protocols were devised: i) a protocol for local specimen procurement, and ii) a protocol for the shipment of whole brain for processing at receiving institute. Both protocols describe a method for collection of CSF and whole brain specimens, including specimen processing for acquisition of frozen and formalin fixed tissue, and specimens for cell culture purposes. The two protocols are discussed in detail below.

### Protocol for Brain Processing and Procurement

Our protocol (Online Resource 1) details specimen processing performed at our center and external sites, where both an autopsy room and pathologist were available. The protocol details procurement and processing of ventricular cerebrospinal fluid (CSF), cerebral hemispheres, and brainstem tissue. Briefly, lateral ventricular CSF is obtained using a sterile needle, and CSF is processed for storage and subsequent molecular analysis. The brainstem along with cerebellum were removed en bloc from the cerebral hemispheres, and these two anatomical specimens are processed as fresh frozen and formalin fixed specimens as described below.

**Fig. 1:**
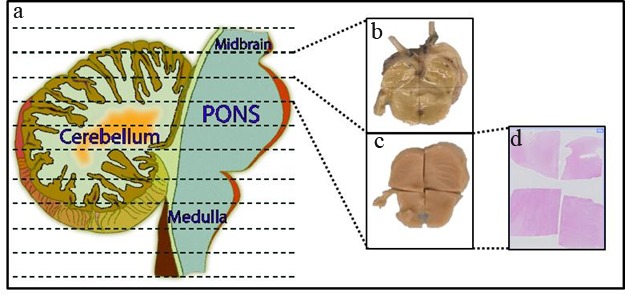
Postmortem processing of brainstem and cerebellum Postmortem brainstem and cerebellum were sectioned into axial sections (a) sectioning through the midbrain, pons and medulla (dotted lines) and through the cerebellum. Alternative sections were either fresh frozen (b) or formalin fixed (c). Fixed samples were then cut into sub-blocks, sections were produced and processed for histological staining (d).

The brainstem is sectioned axially into ten portions starting at the midbrain and ending at the cervicomedullary junction (Fig. 1). Cerebellum is sectioned along with the brainstem. Each section is imaged, measured and alternated for preservation as fresh frozen or formalin fixed specimens. This provides matched formalin fixed specimens for each frozen section, suitable for molecular studies including mRNA, protein, microRNA and DNA analysis. Fixed specimens are embedded in paraffin, sectioned for mounting on slides, and hematoxylin and eosin staining. Consequently, a neuropathologist evaluates these stained slides for histopathological analysis, and tumor grading.

**Fig. 2:**
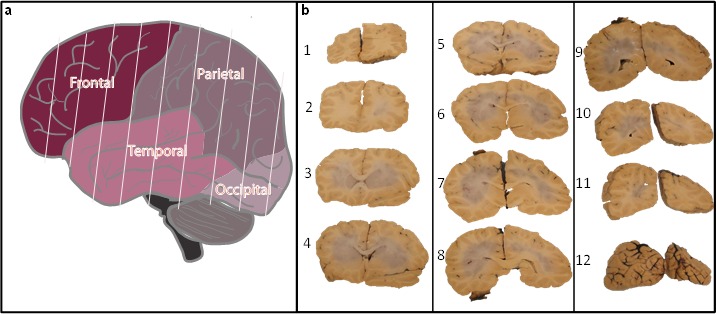
Postmortem processing of whole cortex Upon removal and processing of the brainstem and cerebellum, whole brain was sectioned into coronal slices (a). Sectioning was performed from frontal (section 1, panel b) through the occipital lobe (section 12, panel b) to generate between 10 to 12 slices and imaged for future reference (b). Alternative sections were either frozen or fixed in formalin.

The cerebral hemispheres are also processed in entirety, starting with the frontal lobe and ending at the occipital lobe, producing roughly 12 coronal sections (Fig. 2a). Each section is imaged, and alternative sections are processed for flash freezing or formalin fixation (Fig. 2b).

### Protocol for the shipment of whole brain for processing at receiving institute

We developed a second protocol for cases where tissue procurement at the local site was not possible (e.g. unavailability of pathology team or other resources). This protocol (Online Resource 2) describes specimen (whole brain and CSF) processing for overnight shipment to the receiving institution. The receiving institution can then process the whole brain as described above.

### Standardized Specimen Procurement and Documentation

**Fig. 3:**
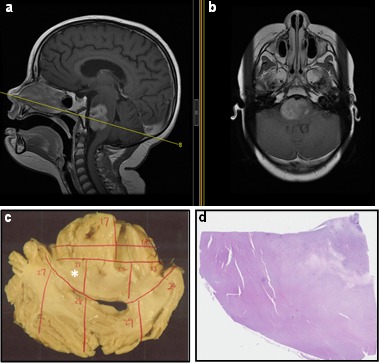
Correlation of histological studies with clinical data Sagittal (a) and axial (b) MR images from a DIPG patient were correlated with autopsied brainstem tissue. The transverse line (a) indicates the plane represented by formalin fixed specimen shown in panel c. Each specimen was clearly labeled (red markings) and cut into sub-blocks. Panel d shows a representative of one sub-block (asterisk in c) that was processed for H&E staining.

All specimens were procured and processed as described in Methods. The procurement method ensured standardization of the autopsy and specimen acquisition process, allowing correlation of autopsied materials with clinical data. For example, due to the accurate characterization and documentation of sections obtained from brainstem, we were able to correlate frozen tumor samples with representative images on MR studies (Fig. 3a, b). Moreover, this standardized specimen procurement method can facilitate specimen tracking in cases where original autopsy material is shared among various institutions (Fig. 3c, d).

### Histological Studies of Cortical Sections Obtained by Standardized Autopsy Procedure

**Fig. 4:**
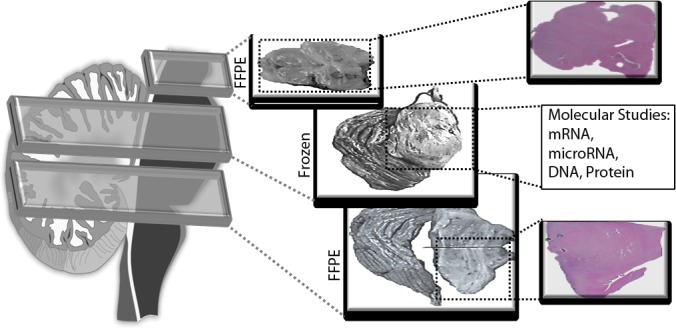
Detailed histological studies of brainstem tissue is enhanced by matched FFPE sections for each frozen specimen The Cerebral cortex and Brainstem were sectioned and each alternative section was processed as FFPE or fresh frozen. Alternative FFPE specimens were stained for H&E and studied by a pathologist. Histopathology of the frozen section was then judged by the histological readings of the two surrounding FFPE samples. Punch cores were then obtained from the middle frozen section for downstream molecular studies including RNA and DNA analyses.

Our standardized autopsy protocol allows for comprehensive molecular characterization of precious DIPG tissue specimens, by ensuring each fresh frozen specimen is also represented by formalin fixed paraffin embedded (FFPE) sections (Fig. 4). Frozen specimens are ideal for molecular analysis including mRNA, protein, and genomic profiling; whereas FFPE samples are more suitable for accurate histological studies. This combination of tissue preparations allows for comprehensive analysis of obtained specimens.

### Generation of murine Model of DIPG Using Postmortem Specimens

**Fig. 5:**
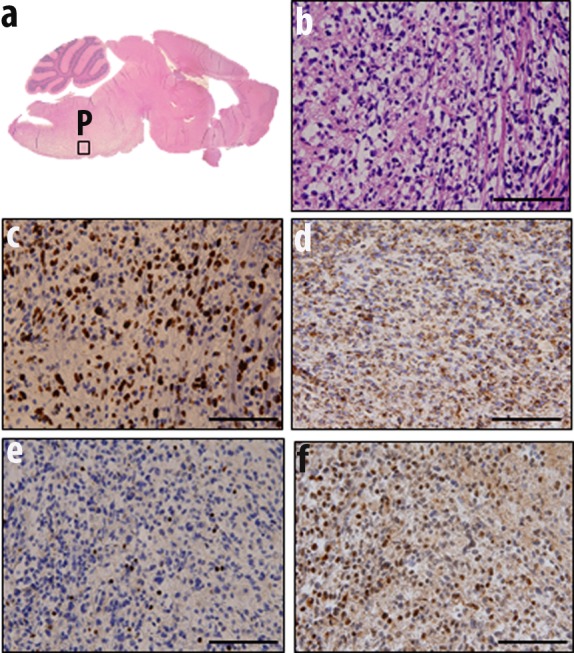
Orthotopic injection of DMSO preserved cells results in diffuse pontine tumors (a) Mice were injected orthotopically in brainstem with cells stored in DMSO. Mice that showed signs of tumor development were sacrificed, brains were fixed and processed for histological studies. H&E staining showed tumor formation in pons (P) as judged by (b) hypercellularity, (c) proliferation marker Ki67, (d) human mitochondrial protein MAB1273 (e) histone 3 K27 trimethylation, and (f) histone 3 K27M mutation.

Fresh autopsy specimens were stored in DMSO as described in Materials and Methods.. DMSO preserved specimens (10 hour post mortem) from one patient were orthotopically injected into the brainstem of ten-day old (p10) mice (n=10). Injections were performed according to established methods [7] by hand at 2 mm posterior to the bregma at the midline position. Seventy percent of injected mice resulted in infiltrating pontine (Fig. 5a, b) or cortical (data not shown) tumors positive for proliferative marker Ki67 (Fig. 5c ). These tumors were also positive for human mitochondrial protein as detected by MAB1273 antibody (Fig. 5d). Tumors showed reduced Histone 3 K27 trimethylation staining (Fig. 5e) but increased Histone 3 K27 mutation (Fig. 5f) indicating their human cell of origin.

## DISCUSSION

Diffuse intrinsic pontine glioma is a fatal childhood cancer with no effective treatment. Surgical resection is not possible, and tissue biopsies are rarely obtained. Postmortem donation is therefore a major source of biological specimens for studying DIPG. Recent identification of Histone 3 K27M mutation and other unique genomic aberrations driving DIPG were made possible by the availability of autopsied and rare biopsied specimens for molecular analysis [2, 4, 5, 8, 9]. Since molecular studies of these specimens are largely affected by procurement procedures, ongoing collaborative efforts to centralize and standardize postmortem DIPG specimen procurement and analysis are underway. Furthermore, we show that up to 73% of donations were initiated by patient's family. This remarkable willingness to donate tissue specimens demonstrates that families in the DIPG community already perceive a potential to improve the understanding of DIPG through tissue donation for research. In light of this observation, it is important to inform all families about the possibility to donate specimens for research, and to emphasize the significant impact this gift can have on accelerating scientific discoveries to improve DIPG care and treatment. Such measures can potentially increase participation in post-mortem donation. In our experience, the optimal time to approach the patient's family for discussing postmortem donation is after completion of radiation treatment, when the patient's clinical symptoms are stabilized. We describe a comprehensive protocol to allow systematic, effective procurement of postmortem DIPG specimens for further molecular studies.

Recent studies of DIPG tissue specimens, and our own observations (unpublished data), suggest tumor extension and local invasion beyond the neuroanatomical confines of the brainstem [10]. We therefore find it prudent to procure and process the brain in its entirety for future comprehensive studies. Recent studies have indicated that WHO-based histological grading does not predict clinical outcome for DIPG [11]. Overall survival of H3.3 K27M mutant DIPG patients with grade II tumors was similar to those with grade IV GBM tumors (also with H3.3 K27M mutation). Comparative studies of biopsied and autopsied brain specimens have shown that despite mRNA and protein degradation, autopsied specimens are a reliable source for biological analysis [12-14]. Such comparisons, however, are not often feasible for DIPG due to the lack of upfront biopsies. However, Zarghooni and colleagues [15] analyzed nine postmortem and two surgical samples and showed no significant differences in gene expression profile across surgical and postmortem specimens. We and others have published molecular studies of postmortem specimens and shown the reliability of these specimens for proteomics, genomics, and mRNA studies [2, 16].

**Fig. 6:**
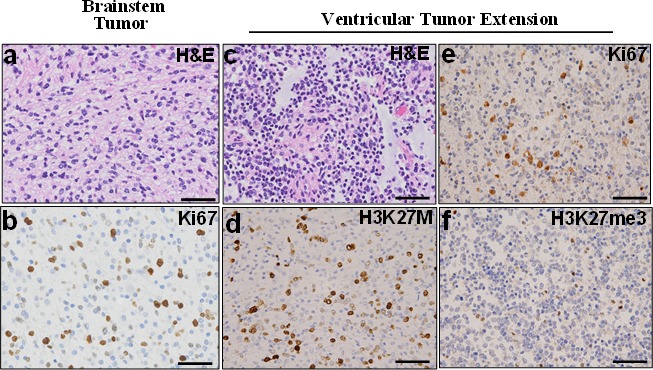
Ventricular extended tumor retains characteristics of primary pontine tumor Histological staining were performed using the primary pontine (a and b) and extended ventricular (c-f) tumor specimens. H&E staining showed highly cellular tumors (WHO IV). Both pontine (b) and extended ventricular (d) tumors were positive for Ki67, (e) while the ventricular retained high expression of histone 3 mutation and (f) reduced histone 3 trimethylation.

Furthermore, DIPG specimens are routinely shared between collaborative institutions. In many instances, these specimens have been labeled as “pontine tumor,” with the precise anatomic tumor location not clearly indicated (e.g. dorsal versus ventral, rostral versus caudal). The precise anatomic origin of acquired specimens is critical for data comparison and validation assays. Our method of imaging each fresh frozen and FFPE specimen facilitates more accurate specimen tracking and characterization to ensure data are generated from tumor regions with similar histological characteristics (Fig. 3c). We found that the xenograft line closely represents primary human tumor by expressing histone 3 K27M mutant phenotype. Expression of human mitochondrial proteins in the murine tumor is further confirmation of the model's fidelity and suitability for preclinical testing (Fig. 5a-f). In light of the recent debates on the extent of DIPG infiltration into surrounding structures, we suggest molecular analysis of mouse models created by orthotopic injection of primary and extended tumors obtained from the same patient. Our postmortem specimen procurement method allows for this, as well as for studying the mechanism of tumor extension. Our preliminary studies show that extended tumor retains the proliferative and histone 3 mutation status of the primary tumor (Fig. 6). However, further studies are prudent to address whether histone 3 K27M mutation is preserved along the path of tumor infiltration in cases of tumor extension, and whether additional mutations are gained along the path of migration.

Another essential question to address is the nature of the direct tumor extension (anatomic continuity) or metastasis (spread to distant sites within the central nervous system) in DIPG. To our best knowledge, currently, there are no published studies establishing whether DIPG spreads by direct extension or metastasis throughout the CNS. The methods proposed here could potentially shed light on this issue, and provide insight into the infiltrative vs. metastatic nature of DIPG.

In summary, we believe that implementation of the protocol described here by the scientific community could standardize the method of acquisition and processing of rare DIPG tumor specimens. Adoption of these methods could facilitate standardized, efficient analysis of these precious specimens across research consortia ensuring optimal utilization. This practice could therefore help to clarify the biological and infiltrative nature of DIPG, and facilitate the standardization of DIPG postmortem studies.

## MATERIALS AND METHODS

### Human Specimen Collection

All local specimens were collected in accordance with Children's National Health System (CNHS) Institutional Review Board (IRB) approvals (IRB# 1339, #463 and #747). Specimens from sites outside CNHS were obtained in accordance to the local IRB approvals. Autopsy consent was obtained in cases where an IRB protocol did not exist, or the decision to donate was made during or after patient's death.

### Specimen Procurement and Processing for Cell Culture and Orthotopic Injection

In order to obtain specimens for cell culture or orthotopic injection, tissue (~0.25cm thick with a surface of ~0.5cm X 0.5cm) from the brainstem and supratentorial cortex were collected into Hibernate A media, which was then transferred on ice to a BSL 2 hood. Tissue was placed in a petri dish containing HBSS-Hepes, and minced into tiny pieces using a sterile No. 10 scalpel. After pelleting by centrifugation (200 g for 5 min), a portion of the tissue pellet was frozen in complete media containing 10% DMSO to use with orthotopic injections at a later date. The remaining pellet was subjected to enzymatic digestion to prepare a single cell suspension. Digestion buffer containing DNase I (250 units/ml) and Collagenase IV (1mg/ml) in HBSS-Hepes was added to the tissue pellet and incubated in 37^°^C water bath for 15 min. Digestion was stopped by diluting the sample with HBSS-Hepes, and cells were pelleted by centrifugation. The cell pellet was then incubated with 1 ml of RBC lysis buffer at 37^°^C for 1 min. RBC digestion was inactivated by adding HBSS-Hepes and cells were collected by centrifugation. The cell pellet was then resuspended in 15 ml of HBSS-Hepes and filtered through a 70 micron cell strainer to remove undissociated tissue and debris. Cells were then counted using trypan blue exclusion assay, and divided into two fractions before processing for cell culture, or orthotopic injection into mice. For culturing, cells were pelleted and plated in Neurobasal A medium containing 50% of DMEM F12 and supplemented with B27 without vitamin A (2%), human-basic FGF (20 ng/mL), human-EGF (20 ng/mL), human PDGF-AB (20 ng/mL), and heparin (10 ng/mL). For immediate orthotopic mice injections cells were collected as a pellet and maintained on ice. For injection of cells from DMSO frozen tissue, samples were thawed quickly and resuspended in HBSS-Hepes. After passing through a 70 μm cell strainer, cells were counted and collected by centrifugation as a pellet followed by orthotopic injection as described below.

### Orthotopic injection of DIPG tumor cells into Scid mice

NOD *SCID* gamma (NSG) mice ranging from 2 to 10 days old were used for orthotopic injection of cells prepared from DIPG postmortem specimens. Mice were subjected to hypothermia-induced anesthesia. Injection site was sterilized, and 1×10^5^ cells were injected into the brainstem (2 mm posterior to the bregma at the midline position) in 2 μl volume using a gas tight Hamilton syringe with a 26 gauge needle [7]. Injected mice were monitored daily, and euthanized after showing symptoms of brain tumor such as ataxia and enlarged head. All procedures performed on mice are in accordance with the CNHS IACUC protocol # 292-12-05.

## SUPPLEMENTARY MATERIALS




