# Supplementary material for: A standardized autopsy procurement allows for the comprehensive study of DIPG biology
Source: Oncotarget. 2015 Jan 24;6(14):12740–7. doi: 10.18632/oncotarget.3374 (PMC4494970; doi:10.18632/oncotarget.3374)
Supplement: Supplementary file 1 [file oncotarget-06-12740-s001.pdf]

A standardized autopsy procurement allows for the comprehensive study of DIPG biology

Supplementary Material 1

Protocol for procuring postmortem brain tissue and CSF

This protocol details the collection and storage procedures for postmortem collection of the following specimens:

A. Ventricular CSF

B. Cortex

C. Brainstem

<u>Detailed Procedure for Sample Collection and Storage:</u>

CSF COLLECTION

1. Expose the brain using the local pathologist's standards and protocols standard autopsy technique.
2. Use a sterile syringe of 18 or 21 gauge needle to collect ventricular CSF. This is best done by gently pulling the hemispheres laterally and aiming the needle laterally from the midline just over the corpus callosum. Extra CSF can also be collected when available from cisterns during brain removal.
3. Collect CSF samples into 2-4ml cryotubes and place on dry ice for immediate shipment or at -80 C for later shipment.

   NOTE: When procedure is done at home institute (or when possible), aliquot CSF into 1.5 ml tubes, centrifuge at 16,000 X g in cold room for 10 minutes to remove any contaminating blood cells. Supernatant should then be collected, aliquoted and stored at -80C.

BRAINSTEM COLLECTION

1. Remove the entire brain, intact using standard autopsy technique.
2. Weigh specimens.
3. Remove the brainstem along with cerebellum from the cerebral hemispheres at its superior-most aspect (level of substantia nigra).

4. Make 5 mm horizontal slices from superior to inferior, leaving brainstem and cerebellum attached (Figure 1). Label the slices 1 through (n).
5. Image slices next to a tape measure.

Fresh Specimen for Cell Culture or intracranial injection Purposes

A. Using sterile conditions, cut a 0.5 cm x 0.5 cm section of tumor and place in sterile cell culture brain tissue preservation and transport media (hibernate A media).
B. Place on ice for immediate shipment to receiving institute or for culturing or intracranial injection.

Frozen Specimens

A. Wrap even numbered slices 2, 4, 6, 8, etc. intact in aluminum foil and snap freeze in liquid nitrogen-cold isopentane as per procedure below. Place in plastic biohazard bags that are labeled with the information such as protocol ID, patient ID and number of horizontal slice, and place on dry ice for shipment or -80˚C for storage.

FFPE Specimens

A. Place remaining odd numbered slices in neutral buffered formalin (10%).
B. After adequate fixation, photograph both slides of each slice of tissue and use these photographs as a map of cassettes submitted for processing.

<u>Cortex Procurement</u>

Cortex procurement will be done as follows:

1. Slice cortex into coronal section from frontal lobe to occipital lobe.
2. Image slices.
3. Freeze or formalin fix alternative slices as described above for brainstem.

Detailed Procedure for freezing tumor and normal specimens

1. Fill a stainless steel beakers halfway with isopentane, place in liquid nitrogen for about 10 minutes and then transfer onto dry ice contained in a cooler.
2. Keep the dry ice cooler covered with a lid throughout the procedure.
3. Prepare 8-10 square pieces of aluminum foil – these should be placed in the cooler of dry ice to cool them before wrapping the brain blocks.
4. Label a biohazard plastic bag clearly as TUMOR ; DATE; and sample ID, and place on dry ice.
5. Label a biohazard plastic bag clearly as NORMAL; DATE; and sample ID, and place on dry ice.
6. Make sure isopentane is well cooled (~20 minutes) and aluminum foil squares are chilled on dry ice.
7. Place each autopsied specimen on a piece of aluminum foil. Using tweezers, hold the foil and submerge the tissue in isopentane. Hold for 15-20 seconds. Remove from isopentane and place on dry ice to allow isopentane to evaporate.
8. Place another cooled aluminum foil on top of the tissue, fold each side,
9. Dip the foil covered tissue in liquid nitrogen and hold for 10 seconds and place in corresponding cooled TUMOR or NORMAL biohazard plastic bags.
10. Place in a box with plenty of dry ice for shipment or in -80˚C freezer for later shipment or storage.

CHECK LIST

Pathologist Name:
Phone Number:

Donor Name:
Autopsy ID:

Fresh Brain Weight (grams):

Time of Death:
Date:

Time Fresh Specimen Collection Started:
Time Autopsy Was Concluded:

	Specimen Collected	Storage	Shipment
☐	Ventricular CSF	Wet ice	Cold Pack
☐	Blood in Purple and Red top tubes		
☐	Brainstem Frozen	-80˚C	Dry Ice
☐	Brainstem FFPE		
☐	Cortex Frozen	-80˚C	Dry Ice
☐	Cortex FFPE		
☐	Fresh Tumor Specimen for Cell Culture	hibernate media	hibernate media

Thank you for helping us learn more about DIPG and helping other children with this diagnosis
