# Supplementary material for: A standardized autopsy procurement allows for the comprehensive study of DIPG biology
Source: Oncotarget. 2015 Jan 24;6(14):12740–7. doi: 10.18632/oncotarget.3374 (PMC4494970; doi:10.18632/oncotarget.3374)
Supplement: Supplementary file 2 [file oncotarget-06-12740-s002.pdf]

A standardized autopsy procurement allows for the comprehensive study of DIPG biology

Supplementary Material 2

Protocol for procuring and shipment of whole brain and CSF

The following supplies should be sent to the autopsy site a head of time for procurement and shipment of CSF and autopsieds whole brain.

- 15mL Falcon tubes (X5)
- Nylon tie wraps
- High density polyethylene (HDPE) container
- Styrofoam box
- Plastic biohazard bags

The following procedure is followed for procurement and shipment of CSF and whole brain

1. Collect CSF from Corpus callosum region into a provided 15mL Falcon tubes and place it in the Styrofoam container.
2. Harvest fresh whole brain intact and place in double plastic bags. Seal each bag securely with the tie wraps provided.
3. Place the bag containing whole fresh brain in to a HDPE container.
4. Add ice cold water to the container, place the lid firmly and seal it.
5. Place the sealed HDPE container in to a plastic bag lined styrofoam box filled with wet ice and seal securely.
6. Ship the package via FedEx same-day delivery service (where available) or priority overnight service.

7. Provide the receiver with the tracking number.
